# Characterizing acetogenic metabolism using a genome-scale metabolic reconstruction of *Clostridium ljungdahlii*

**DOI:** 10.1186/1475-2859-12-118

**Published:** 2013-11-25

**Authors:** Harish Nagarajan, Merve Sahin, Juan Nogales, Haythem Latif, Derek R Lovley, Ali Ebrahim, Karsten Zengler

**Affiliations:** 1 Department of Bioengineering, University of California San Diego, La Jolla, CA, USA; 2 Department of Microbiology, University of Massachusetts, Amherst, MA, USA

## Abstract

**Background:**

The metabolic capabilities of acetogens to ferment a wide range of sugars, to grow autotrophically on H_2_/CO_2_, and more importantly on synthesis gas (H_2_/CO/CO_2_) make them very attractive candidates as production hosts for biofuels and biocommodities. Acetogenic metabolism is considered one of the earliest modes of bacterial metabolism. A thorough understanding of various factors governing the metabolism, in particular energy conservation mechanisms, is critical for metabolic engineering of acetogens for targeted production of desired chemicals.

**Results:**

Here, we present the genome-scale metabolic network of *Clostridium ljungdahlii,* the first such model for an acetogen. This genome-scale model (iHN637) consisting of 637 genes, 785 reactions, and 698 metabolites captures all the major central metabolic and biosynthetic pathways, in particular pathways involved in carbon fixation and energy conservation. A combination of metabolic modeling, with physiological and transcriptomic data provided insights into autotrophic metabolism as well as aided the characterization of a nitrate reduction pathway in *C. ljungdahlii*. Analysis of the iHN637 metabolic model revealed that flavin based electron bifurcation played a key role in energy conservation during autotrophic growth and helped identify genes for some of the critical steps in this mechanism.

**Conclusions:**

iHN637 represents a predictive model that recapitulates experimental data, and provides valuable insights into the metabolic response of *C. ljungdahlii* to genetic perturbations under various growth conditions. Thus, the model will be instrumental in guiding metabolic engineering of *C. ljungdahlii* for the industrial production of biocommodities and biofuels.

## Background

Acetogenic microorganisms have unique metabolic capabilities that, if understood, could be harnessed to greatly increase strain engineering design options for microbial production of biofuels and biocommodities. Acetogens were discovered for their ability to autotrophically reduce CO_2_ to acetate and conserve energy simultaneously using the Wood-Ljungdahl pathway [1]. In addition to reducing CO_2_, acetogens can ferment a wide variety of sugars as well. Their ability for autptrophy allows them also to grow on synthesis gas (H_2_/CO/CO_2_) by utilizing either H_2_/CO_2_ or CO alone. More recently, acetogens like *Sporomusa ovata* and *Clostridium ljungdahlii* have been shown to be capable of another form of autotrophic metabolism, called microbial electrosynthesis [2]. Microbial electrosynthesis is a process in which microorganisms directly use electric current to reduce carbon dioxide to multi-carbon organic compounds that are excreted from the cells into extracellular medium [3]. These discoveries further expand the range of economically viable feedstocks that can be used for industrial production of biofuels and biochemicals [4].

However, to efficiently engineer acetogens into platform strains and production hosts for chemicals at an industrial scale, a thorough understanding of the metabolic capabilities and aspects of energy conservation is a necessary prerequisite. The Wood-Ljungdahl pathway of carbon fixation employed by acetogens is believed to be one of the most ancient metabolic pathways [5]. Physiological and biochemical aspects governing this metabolic capability have been poorly characterized with several fundamental discoveries being made only recently [6,7]. One of the fundamental characteristics of acetogenic metabolism discovered recently is the concept of flavin-based electron bifurcation. In this mechanism, there is a concomitant coupling of an endergonic redox reaction with the oxidation of the same electron donor with higher potential electron acceptors. This feature is believed to play a key role in the energy conservation mechanisms of acetogens [8].

Constraints based reconstruction and analysis (COBRA) has been a powerful technique for discovering and understanding new capabilities and content in microorganisms, as well as in guiding metabolic engineering efforts for targeted production [9]. The COBRA approach relies on a genome-scale metabolic network reconstruction, which enumerates the metabolic transformations and the genes encoding them in a mathematical format. This reconstructed network together with physiological data can then enable the prediction of the functionality of an organism under conditions of interest. Such a validated and accurate network can be utilized for prospective design and engineering of cellular networks [10]. Furthermore, genome-scale metabolic networks of bacteria and constraints-based modeling have been instrumental in guiding metabolic engineering at an industrial scale [11].

In this study, we reconstructed the first genome-scale metabolic network of an acetogen, *Clostridium ljungdahlii*. We characterized the metabolic phenotypes of this bacterium under heterotrophic and autotrophic growth conditions. We further utilized physiological and transcriptomic data to elucidate novel biological capabilities and aspects of energy conservation during autotrophic metabolism of *C. ljungdahlii*.

## Results and discussion

### Genome-scale reconstruction of the acetogen *Clostridium ljungdahlii*

**Figure 1:**
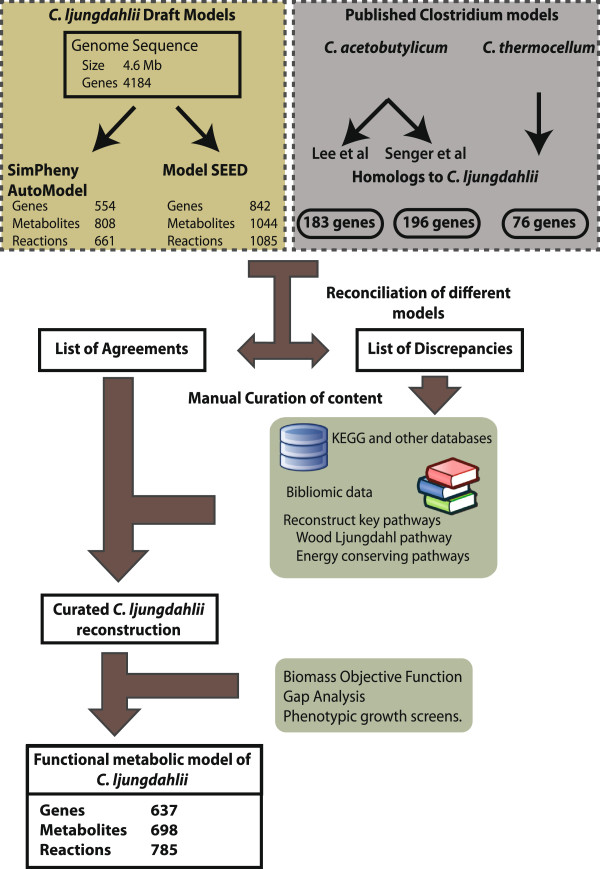
**Reconstruction workflow.** Iterative reconciliatory reconstruction workflow adopted for generating the metabolic model for *C. ljungdahlii*.

The genome-scale metabolic network for *C. ljungdahlii* was reconstructed using a four-step integrative reconciliatory workflow involving four published models of related clostridia species and two draft models (Figure 1). The first draft metabolic model was generated based on the *C. ljungdahlii* genome annotation [12] using the AutoModel functionality of SimPheny (Genomatica, San Diego), while the second draft model was generated using the ModelSEED database [13]. In addition to these two draft models, homologs to *C. ljungdahlii* genes were identified in published genome-scale reconstructions of related clostridia species (*C. acetobutylicum, C. thermocellum,* and *C. beijerenckii*) [14-17] using the Smith-Waterman alignment. A 60% amino acid sequence identity cutoff was used to identify *C. ljungdahlii* homologs in the other clostridia genomes. The reactions corresponding to these genes in the respective clostridia models were compiled and reconciled with the two draft models. The list of discrepancies pertaining to nomenclature between the different databases and gene-reaction associations was manually curated with the aid of biochemical literature and databases such as KEGG [18] and SEED [13]. Manual evaluation of new content from the annotation and existing genome-scale reconstructions consisted of gathering genetic, biochemical, sequence, and physiological data and reconciling this information to determine the likelihood of each reaction being present in the organism. The curated reconstruction was evaluated for functional performance with the aid of a biomass objective function that was formulated using an existing template (see Methods). Using inference based on pathway function, as well as the SMILEY computational algorithm [19,20], which predicts reactions that fill gaps in a metabolic network, reaction content was added to the network so that it could produce the necessary biomass components. This resulted in a final network (iHN637) consisting 637 genes, 785 reactions, and 698 metabolites. This iHN637 reconstruction represents the first genome-scale metabolic model of an acetogen.

### Functional testing of the model

The functional capabilities of the iHN637 reconstruction were evaluated by simulating growth under different conditions using the developed biomass objective function (Methods). iHN637 was successfully able to simulate heterotrophic growth on several of the known substrates [21] (fructose, glucose, gluconate, arabinose, ribose, xylose, ethanol, formate, serine, citrulline, pyruvate, arginine, aspartate, and glutamate). Moreover, autotrophic growth with H_2_ and CO_2_ as well as just CO alone was feasible using the iHN637 reconstruction. The predicted growth rates and acetate production rates for each of these various substrates are provided in Table 1. In addition to acetate and ethanol, the iHN637 metabolic model is also capable of producing lactate and 2,3-butanediol using native pathways as described elsewhere [22]. Furthermore, the model was able to successfully predict a growth rate consistent with experimental observations for heterotrophic growth on fructose. When the iHN637 model was constrained with experimentally determined uptake rates of fructose (1.8 mmol/gDWh), the growth rate predicted by the model (0.077 h^-1^) was in good agreement with the experimentally measured growth rates (0.072 h^-1^). The simulated acetate production rate (2.7 mmol/gDWh) was also consistent with the measured value of 2.85 mmol/gDWh (Additional file 1: Figure S1).

**Table 1:** Model predicted growth rates and acetate production rates using iHN637

**Substrate**	**Substrate uptake rate (mmol/gDW/h)**	**Specific growth rate (1/h)**	**Acetate production rate (mmol/gDW/h)**
**Fructose**	1.88	0.077	2.70
**Fructose**	5	0.224	9.30
**Glucose**	5	0.212	9.61
**Gluconate**	5	0.191	8.91
**Arabinose**	5	0.174	8.09
**Ribose**	5	0.133	9.12
**Xylose**	5	0.174	8.09
**Formate**	5	0.011	0.96
**Serine**	5	0.074	4.36
**Citrulline**	5	0.034	4.14
**Pyruvate**	5	0.063	4.65
**Arginine**	5	0.035	4.13
**Asparatate**	5	0.097	5.05
**Glutamate**	5	0.108	8.51
**CO**_**2**_**(H**_**2**_**)**	10(20)	0.034	4.14
**CO**	20	0.06	3.48

The substrate uptake rates listed in the table was used to constrain the model.

### Characterization of a nitrate reduction pathway in *Clostridium ljungdahlii*

**Figure 2:**
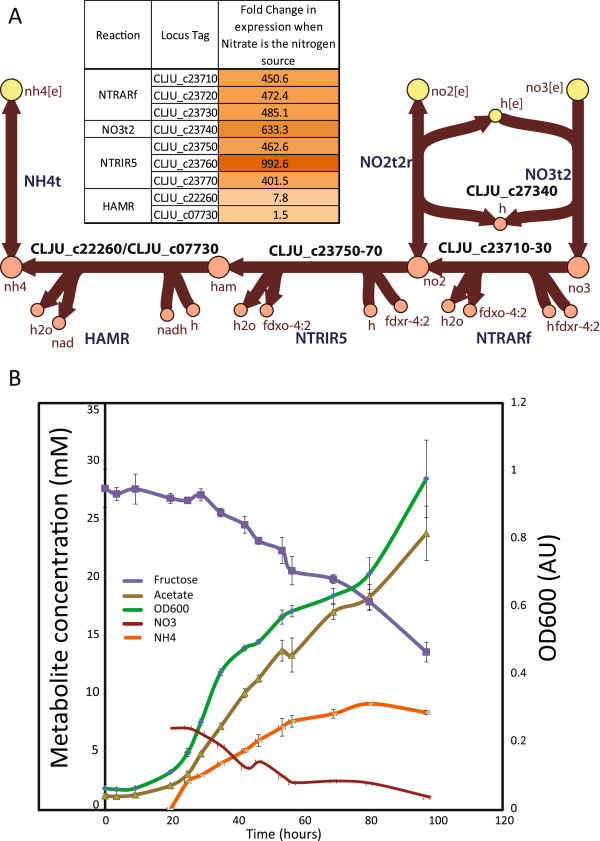
**Characterizing the nitrate reduction pathway in *C. ljungdahlii. *(A)** Pathway map of the reconstructed nitrate reduction pathway with the respective genes associated to the reactions. Inset shows the differential expression of the genes in the nitrate reduction pathway under ammonium-free medium with nitrate compared to nitrate-free medium with ammonium as the nitrogen source. Reaction abbreviations: NO3t2 (nitrate transporter), NO2t2r (nitrite transporter), NTRARf (nitrate reductase), NTRIR5 (nitrite reductase), HAMR (hydroxylamine reductase), NH4t (ammonium transporter). **(B)** Physiological data when *C. ljungdahlii* is grown on fructose in ammonium-free media with nitrate as the nitrogen source. Purple line represents fructose, green line represents OD_600_, brown line represents acetate, red line represents nitrate and orange line represents ammonia measurements respectively.

A novel nitrate reduction pathway for clostridia was reconstructed in the iHN637 model based on the *C. ljungdahlii* genome annotation. In addition to the glutamine synthetase and glutamine:2-oxoglutarate aminotransferase or the fixation of dinitrogen using a molybdenum-dependent nitrogenase, this pathway could serve as a third possible nitrogen assimilation route in *C. ljungdahlii*. This pathway presents characteristics of both, assimilatory and respiratory nitrate reductases and the reduction of nitrate to ammonia via hydroxylamine resembles the nitrate respiratory system recently identified in *Nautilia profundicola*[23]. The nitrate reduction pathway consists of a soluble nitrate reductase (NTRARf), a nitrite reductase (NTRIR5), and an additional hydroxylamine reductase (HAMR) (Figure 2A). Transcriptomic profiling of *C. ljungdahlii* grown heterotrophically on fructose in medium containing ammonium or ammonium-free medium with nitrate as a nitrogen source, helped validate this pathway. The genes encoding for the nitrate reduction pathway were all significantly upregulated (>8-fold) when nitrate was provided in ammonium-free medium. Specifically, the genes encoding for nitrate reductase, nitrite reductase, and the nitrate transporter were upregulated greater than 400-fold (Figure 2A). Growth experiments showed that nitrate was consumed with stoichiometric production of ammonium (Figure 2B), further validating the proposed pathway. Furthermore, modeling simulations predict a 15% reduction in acetate flux when nitrate served as nitrogen source due to nitrate reduction acting as an additional electron sink (Additional file 2: Table S1). The diversion of electrons seems to impact the acetate production gained through fixation of CO_2_ using electrons obtained from fermenting fructose. In addition, nitrate reduction has been thought of as a primitive mode of energy metabolism in certain clostridia with potential roles in balancing electrons and affecting growth yields during fermentation [24]. The identification of this pathway provides the opportunity for a detailed investigation on its potential functional effects on the fermentation of *C. ljungdahlii*.

### Reconstructing the carbon fixation pathway in *C. ljungdahlii*

The *C. ljungdahlii* genome encodes for all the genes involved in the Wood-Ljungdahl pathway of carbon fixation [12]. Accordingly, this pathway was incorporated into the iHN637 reconstruction. The pathway consists of two different branches, one CO_2_ contributing to the methyl group of acetyl CoA via the eastern branch and the other CO_2_ contributing to the carbonyl group of the acetyl CoA via the western branch [25]. The key enzyme in this pathway is the CODH/ACS complex, which performs the dual activity of reducing CO_2_ to CO via the carbon monoxide dehydrogenase (CODH) activity and the subsequent formation of acetyl CoA through the acetyl-CoA synthase (ACS). It has been suggested that the CODH/ACS complex functions in a manner that CO resulting from CODH activity is kept as a bound metabolite in the complex to diminish thermodynamic barriers involved in this energy intensive process [26]. Furthermore, the genome of *C. ljungdahlii* suggested that CO oxidation to CO_2_ is likely to be catalyzed by a different carbon monoxide dehydrogenase gene (CLJU c09090-09110) other than the ones encoded by the CODH/ACS complex. Taking these into consideration, the iHN637 reconstruction incorporates the CODH/ACS reaction as the net reaction of carbon monoxide dehydrogenase and acetyl CoA synthase activity, and associates the gene cluster CLJU c09090-09110 to the reaction representing CO oxidation to CO_2_. The methylenetetrahydrofolate dehydrogenase reaction catalyzed by the bifunctional FolCD (CLJU_c37630) was assumed to be NADPH-dependent based on sequence similarity with the corresponding enzyme in the acetogen *Moorella thermoacetica*[25,27]. The energy conservation mechanisms associated with autotrophic growth using the Wood-Ljungdahl pathway is discussed in detail in the following section.

### Analysis of energy conservation steps in *C. ljungdahlii*

**Figure 3:**
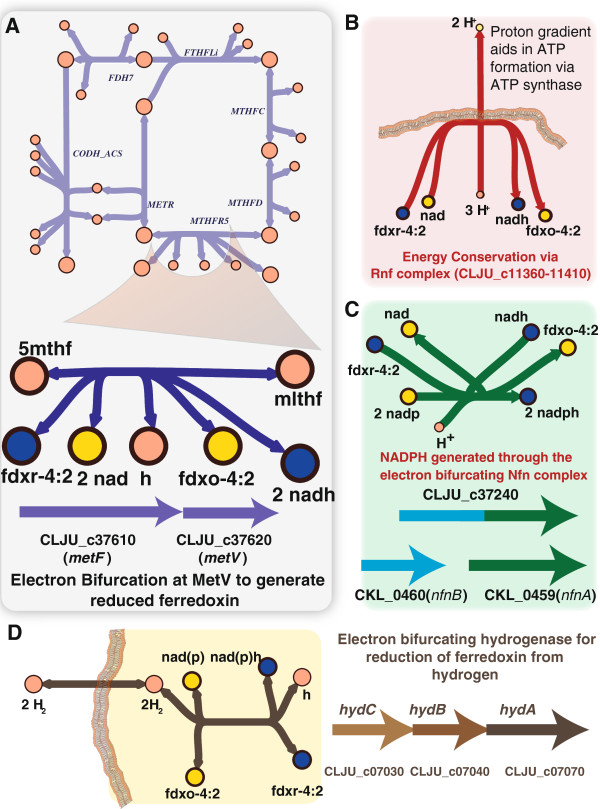
**Energy conservation mechanisms in *C. ljungdahlii. *(A)** Shown is the Wood-Ljungdahl pathway with an emphasis on the electron bifurcation in the Wood-Ljungdahl pathway at the methylene-tetrahydrofolate deydrogenase small subunit to generate reduced ferredoxin. The detailed Wood-Ljungdahl pathway is shown in Figure 4. Reaction abbreviations are the same as those in Figure 4. **(B)** Energy conservation through proton gradient established at the Rnf complex. **(C)** Identification of a gene encoding the electron bifurcating transhydrogenase for the generation of NADPH during autotrophic growth (Nfn complex). **(D)** Electron bifurcating hydrogenase for the reduction of ferredoxin from hydrogen during autotrophic growth. Yellow nodes indicate the oxidized form of reducing equivalents and blue nodes indicate the reduced form.

Acetogens have long been thought to be living at the thermodynamic limit due to the energy requirements of the Wood-Ljungdahl pathway [7]. Given that the ATP generated from acetate production is required for the activation of formate, it has been proposed that acetogens should have additional energy conservation mechanisms [7]. Acetogens have generally been classified into those containing respiratory cytochromes that establish a proton gradient (e.g., *M. thermoacetica*) and those that do not. Organisms such as *Acetobacterium woodii* have been shown to employ a sodium gradient as an energy conservation mechanism [7]. The *C. ljungdahlii* genome sequence has revealed that it falls into a third class of acetogens that neither uses respiratory cytochromes nor a sodium gradient for energy conservation [12]. As reviewed recently, flavin-based electron bifurcation is expected to serve as an alternative mechanism of energy conservation in acetogens [8]. Analysis of the iHN637 reconstruction of *C. ljungdahlii* reveals that electron bifurcation and proton translocating ferredoxin oxidation are critical mechanisms for energy conservation during autotrophic growth in *C. ljungdahlii* (Figure 3). The iHN637 model predicted proton translocation by the membrane-bound Rnf complex while oxidizing ferredoxin and reducing NAD was essential for autotrophic growth. Specifically, the proton gradient established by the Rnf complex is essential for driving ATP generation through ATP synthase (Figure 3B). This prediction is consistent with recent experimental observations of the lack of autotrophic growth in a strain where the Rnf complex was deleted [28].

An electron-bifurcating transhydrogenase (Nfn complex) that couples exergonic reduction of NADP with ferredoxin to drive the endergonic reduction of NADP with NADH had been characterized in *C. kluyveri*[29]. Recently, a similar electron bifurcating NADPH transhydrogenase activity was also reported in the acetogen *M. thermoacetica*[30]. A homolog to the *nfnA and nfnB* genes in *C. kluyveri* was identified in the *C. ljungdahlii* genome. This gene (CLJU_c37240) was previously annotated as glutamate synthase. Another recent report describes a similar electron-bifurcating NADPH transhydrogenase activity in *C. autoethanogenum* and further indicates the presence of a homolog in *C. ljungdahlii*[31]. As part of iHN637, this gene (CLJU_c37240) has been assigned the electron bifurcating transhydrogenase reaction (Figure 3C). Simulatons further reveal that this reaction is essential for the interconversion between NADPH and NADH during autotrophic growth.

It was speculated that the highly exergonic reduction of methylene-tetrahydrofolate by NADH could be a site for electron bifurcation to generate additional reduced ferredoxin from NADH [12]. Investigation of the *A. woodii* genome revealed that the small subunit of the methylene-tetrahydrofolate reductase (MetV) can act as a flavoprotein that could aid in this proposed electron bifurcation, thereby indirectly establishing a proton gradient through the Rnf complex [7]. A homolog to the MetV gene from *A. woodii* was identified next to the MetF gene in *C. ljungdahlii* and investigation of the intergenic spacing in the genome revealed a possible organization of MetV and MetF in the same operon (CLJU c37610 and CLJU c37620). Hence, an electron bifurcating methylene-tetrahydrofolate reductase reaction was included in the iHN637 reconstruction (Figure 3A).

### Modeling of heterotrophic and autotrophic metabolism

Transcriptome profiling of *C. ljungdahlii* when grown on fructose and autotrophically on H_2_/CO_2_ identified 114 differentially expressed genes (50 upregulated and 64 downregulated in autotrophic conditions) (Additional file 3: Table S2). Among the key genes that were downregulated during autotrophic growth were genes involved in fructose metabolism, pyrimidine biosynthesis, and amino acid biosynthesis. The downregulation of biosynthetic genes in autotrophic conditions is likely due to the slower growth rate during autotrophic growth compared to heterotrophic growth. Among the key genes upregulated was the *cooS1* (CODH) gene. The *cooS1* gene in *Carboxydothermus hydrogenoformans* is hypothesized to play a role in facilitating electron transfer through the hydrogenase [32]. Hence it is possible that this gene in *C. ljungdahlii* has a similar role while growing on hydrogen. Importantly, transcriptome profiling during autotrophic growth revealed that the gene annotated as *metV* was co-transcribed along with *metF*, further validating the annotation and assignment of its function for electron bifurcation with *metF* (Additional file 4: Figure S2). It must also be noted that genes encoding the Wood-Ljungdahl pathway are not differentially expressed between the conditions examined in this study (heterotrophic growth on fructose versus autotrophic growth on H_2_/CO_2_). This is probably due to the fact that the Wood-Ljungdahl pathway is employed for fixation of CO_2_ using electrons obtained from fermenting sugars [1]. However, a recent transcriptomic study on syngas utilization in *C. ljungdahlii* showed upregulation of the genes encoding the Wood-Ljungdahl pathway under autotrophic growth (4:1 CO:CO_2_) [33]. This difference can be attributed to the differences in the autotrophic conditions investigated in both these studies with one being CO_2_/H_2_ and the other being a condition rich in CO.

**Figure 4:**
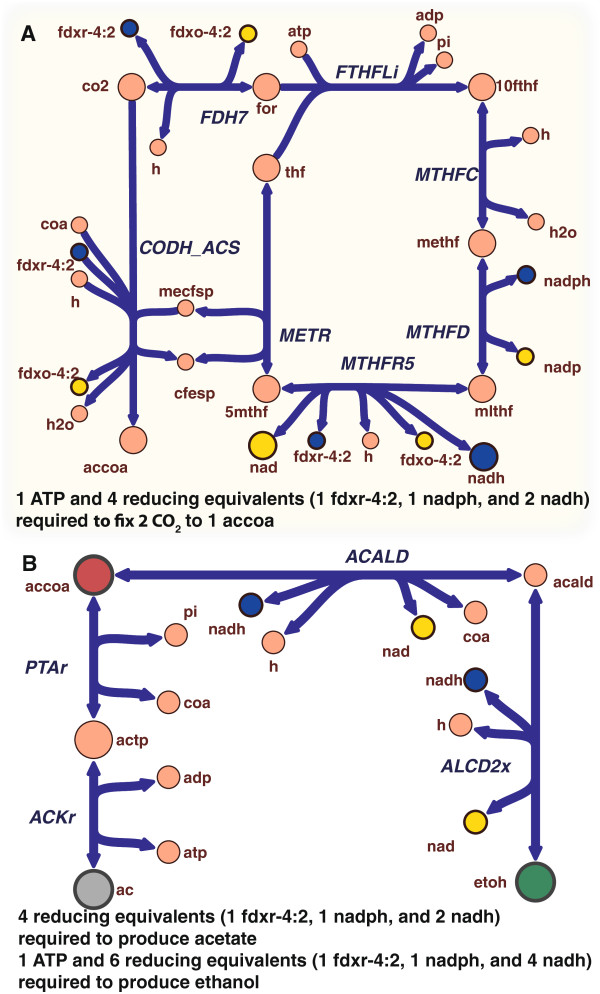
**Wood-Ljungdahl pathway and pathways for acetate and ethanol formation. (A)** Shown is the detailed representation of the Wood-Ljungdahl pathway explicitly summarizing the reducing equivalents required for carbon fixation. **(B)** Pathway showing acetate formation and ethanol formation from acetyl-coA. Reaction abbreviations: FDH7 (formate dehydrogenase), FTHFLi (formate-tetrahydrofolate ligase), MTHFC (methenyltetrahydrofolate cyclohydrolase), MTHFD (methylenetetrahydrofolate dehydrogenase (NADP)), MTHFR5 (5,10-methylenetetrahydrofolate reductase (Ferredoxin) Electron bifurcating), METR (Methyltetrahydrofolate:corrinoid/iron-sulfur protein methyltransferase), CODH_ACS (CODH/Acetyl-CoA synthase), PTAr (phospotransacetylase), ACKr (acetate kinase), ACALD (acetaldehyde dehydrogenase), ALCD2x (ethanol dehydrogenase). Yellow nodes indicate the oxidized form of reducing equivalents and blue nodes indicate the reduced form.

The genome-scale model of *C. ljungdahlii* has revealed that flavin-based electron bifurcation is found to play a critical role in the essential reactions for autotrophic growth, thereby providing an explicit account of the requirement of reducing equivalents for the fixation of CO_2_ to acetyl CoA (Figure 4A). Another instance of electron bifurcation in the autotrophic growth of *C. ljungdahlii* was observed in the first step of H_2_ activation to reduce ferredoxin. A bifurcating hydrogenase (HydABC), which couples the exergonic reduction of NADH from H_2_ to drive the endergonic reduction of ferredoxin from H_2_ has been identified to perform this process in *A. woodii*[7,34]. A homolog to this gene cluster has been identified in *C. ljungdahlii* (Figure 3D). However, in another recent report, a similar electron-bifurcating hydrogenase that is specific to NADPH has been identified and characterized in *C. autoethanogenum*, an acetogen closely related to *C. ljungdahlii*[31]. This report also mentions that the hydrogenase of *C. ljungdahlii* is homologous to the NADP-specific hydrogenase in *C. autoethanogenum*. Since there is no definitive biochemical data available for *C. ljungdahlii* the metabolic reconstruction (iHN637) has both the NAD-specific and NADP-specific electron bifurcating hydrogenases associated to this gene cluster, with the NADP-specific one being set as the default.

**Figure 5:**
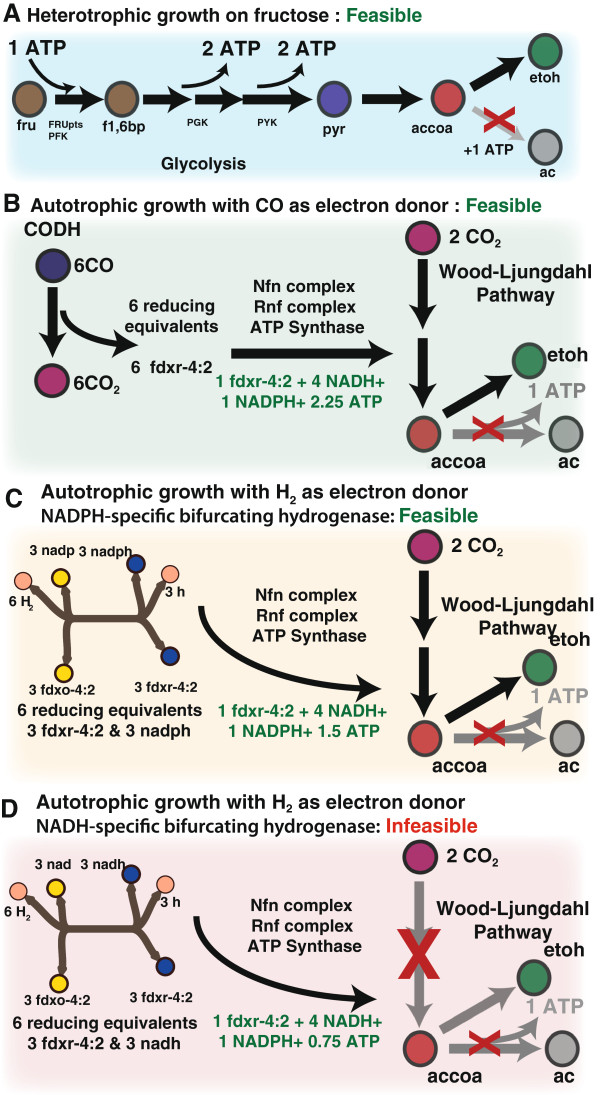
**Insights from modeling genetic perturbation into aspects of energy conservation in *C. ljungdahlii. *(A)** Deletion of acetate kinase during growth on fructose shifts the flux from acetate towards ethanol. **(B)** Growth is feasible when CO is the electron donor despite the deletion of acetate kinase. **(C)** Growth is feasible when H_2_ is the electron donor despite the deletion of acetate kinase if the bifurcating hydrogenase is NADPH dependent. **(D)** Acetate kinase is essential for autotrophic growth with H_2_ as the electron donor if the bifurcating hydrogenase is NADH dependent. Modeling analysis reveals an insufficient ATP in the absence of *ackA* if the hydrogenase is NADH dependent (0.75 ATP) . However, if the bifurcating hydrogenase is NADP dependent, sufficient ATP (1.5 ATP) is generated to enable ethanol production in the absence of *ackA*.

The impact of this cofactor specificity on the metabolic network and the importance of detailing these energy conservation mechanisms was further highlighted when genetic perturbations were modeled under heterotrophic and autotrophic conditions. Specifically, the effect of knocking out acetate kinase (*ackA*) was modeled under heterotrophic growth on fructose and autotrophic growth under the major constituents of syngas (CO alone as well as H_2_/CO_2_) (Figure 5). The deletion of *ackA* is one of the primary targets for optimizing ethanol production in acetogens.

Modeling simulations revealed that the activity of acetate kinase was not essential for growth on fructose. As expected, the lost ATP from the acetate kinase reaction adversely impacted the growth resulting in a 20% reduction in growth yield with the model predicting a diversion of flux to ethanol (Figure 5A).

When ethanol is the desired product from carbon fixation through the Wood-Ljungdahl pathway, 2 reducing equivalents in the form of NADH are required in addition to the 4 reducing equivalents (1 NADPH, 1 ferredoxin, and 2 NADH) and 1 ATP (Figure 4B). Since ethanol production from syngas is an industrially relevant process, iHN637 was used to simulate the effect of this important deletion (*ackA*) under two different autotrophic growth conditions (CO alone as well as H_2_/CO_2_) that reflect the composition of syngas.

Simulations of the acetate kinase knock out under these conditions showed that the essentiality of *ackA* gene was dependent on the source of electrons (CO or H_2_) and the cofactor specificity of the bifurcating hydrogenase. When CO is the sole electron donor, all 6 required reducing equivalents are obtained solely in the form of low-potential reduced ferredoxin. Hence, flux is driven through the Rnf complex to get the necessary NADH for the Wood-Ljungdahl pathway and ethanol formation. Since the Rnf complex couples proton translocation with the oxidation of ferredoxin, this results in the formation of the required ATP to fix CO_2_ using the Wood-Ljungdahl pathway (Figure 5B).

When H_2_ is the electron donor, and the bifurcating hydrogenase is NADP dependent, the 6 reducing equivalents are obtained in an equimolar split of NADPH and ferredoxin (Figure 5C). The Nfn complex ensures conversion of 2 NADPH to 1 NADH and 1 reduced ferredoxin. However, to get the required balance of ferredoxin and NADH, there is a requirement of flux through the proton translocating Rnf complex that interconverts ferredoxin and NADH. While converting 3 ferredoxin to 3 NADH, the proton motive force at the Rnf complex enables the generation of 1.5 ATP. This ensures that carbon fixation and ethanol production is feasible in the absence of *ackA*. However, if the bifurcating hydrogenase is NADH dependent, the required 6 reducing equivalents from this reaction are obtained in the form of 3 NADH and 3 ferredoxin (Figure 5D). The Nfn complex and Rnf complex ensure the interconversion of these reducing equivalents to obtain the necessary NADPH and NADH. However, due to the lower demand for NADH in this scenario, the flux through the proton-translocating Rnf complex is reduced, resulting in the generation of only 0.75 ATP.

This ATP generation due to the lower flux through Rnf complex explains the infeasibility of autotrophic growth of the *ackA* mutant on H_2_/CO_2_ if the cofactor specificity of the bifurcating hydrogenase is NADH. Similarly, since H_2_ is a major component of syngas along with CO, this impact of the cofactor specificity of the bifurcating hydrogenase on energy conservation during autotrophic growth is fundamental to engineering acetogens for the production of desired compounds from syngas.

## Conclusions

This study presents the first genome-scale metabolic network for an acetogen. The model is predictive of the metabolic capabilities of *C. ljungdahlii* and together with the recently developed genetic system [35] can aid in the characterization of its metabolic phenotype and guide engineering strategies. For instance, *C. ljungdahlii* is an important organism in the conversion of synthesis gas. Eliminating acetate production is typically the first choice when engineering acetogens like *C. ljungdahlii* for the production of chemicals like ethanol. Our results highlight the genetic and energetic constraints while rerouting production of target molecules away from acetate. Therefore, engineering strategies have to account for the appropriate cofactor dependencies and the specific energy conservation mechanisms employed depending on the electron source. Furthermore, *C. ljungdahlii* is also capable of microbial electrosynthesis, thereby fixing CO_2_ using electrons directly provided by an electrode [2]. This metabolic model can be extended to obtain insights into potential electron transfer components involved in microbial electrosynthesis. Modeling microbial electrosynthesis would require a systematic evaluation of the different electron transfer pathways through potential carriers such as cytochromes, quinones, or flavins. So far none of these redox active molecules have been identified in *C. ljungdahlii*. Preliminary studies using a quinone extraction procedure and LC-MS have hinted at the presence of a menaquinone-like derivative in *C. ljungdahlii*. While beyond the scope of this study, a thorough investigation of the structure of this molecule and its potential role in electron transfer could significantly aid modeling and characterization of microbial electrosynthesis. The model could also serve as the basis for gaining further insight into the metabolism of other acetogens. Given the different energy conservation mechanisms adopted by acetogens like *M. thermoacetica,* the model enables a systematic comparison of the functional impact of these different mechanisms on acetogenic metabolism in general. Overall, these representative examples emphasize how the detailed metabolic reconstruction of an acetogen can aid in the discovery of new function and guiding strain-design strategies for advanced bioproduction from waste streams.

## Methods

### Reconstruction process

The genome-scale metabolic network for *C. ljungdahlii* was reconstructed using a four-step integrative reconciliatory workflow involving four published models of related Clostridia species and two draft models. The first draft metabolic model was generated using the ModelSEED database [13]. This draft model is generated automatically in a high-throughput manner by comparing the existing genome annotations in the SEED database. To complement this, we generated another automated draft model that relies on a database of curated genome-scale models. This was done using the AutoModel functionality of SimPheny (Genomatica, San Diego). In addition to these two draft models, homologs to *C. ljungdahlii* genes were identified in published genome-scale reconstructions of related Clostridia species (*C. acetobutylicum*[14,15], *C. thermocellum*[16], and *C. beijerenckii*[17]) using the Smith-Waterman alignment. A 60% amino acid sequence identity was employed to identify *C. ljungdahlii* homologs in the other Clostridia genomes. The final curated reconstruction was built in the SimPheny platform (Genomatica, San Diego) to ensure that the reactions in the network were all elementally and charge balanced.

### Generation of the biomass objective function

The Biomass Objective Functions for *C. ljungdahlii* was formulated using a previous template [36]. The protein content was determined to be 43% and for the rest of the macromolecular breakdown, the biomass content previously determined for other Gram-positive bacteria, *B. subtilis* and *C. beijerenckii*, was used [17,37]. However, for modeling teichoic acid composition, the distribution based on *S. aureus* was used due to similarity in terms of low G + C content [38]. It should be noted that prediction of growth rate and unmeasured uptake rates are relatively insensitive to realistic variations in biomass macromolecular weight fractions [39].

### Flux balance analysis simulations

The reconstructed metabolic network was represented in a mathematical format in a stoichiometric matrix S, where the rows correspond to the metabolites and columns correspond to the reactions in the network. Flux Balance Analysis simulations were carried out as described previously [40] using the COBRA Toolbox [41] and the SimPheny framework (Genomatica, Inc., San Diego, CA) was used for simulations. The objective used in the simulations was maximizing growth through the biomass objective function. *In silico* simulations of gene knockout was carried out using the "singleGeneDeletion" function of the COBRA toolbox [41]. The metabolic models for simulating heterotrophic and autotrophic growth are available for download (Additional file 5: Model 1 and Additional file 6: Model 2).

### Bacterial growth conditions and chemical analysis

*Clostridium ljungdahlii* (ATCC 55383) was grown in 125 mL serum bottles under anaerobic conditions containing 100 mL of PETC medium (ATCC medium 1754) at 37°C. For growth experiments with nitrate, 1 mL of an anoxic NaNO_3_ stock solution (1 M) was added to the medium. Ammonium was omitted in some of the experiments from the medium containing nitrate. Growth was routinely determined by measurement of the OD_600_. Hydrogen and other gases in the cultures were quantified using gas chromatography as previously described [42]. Concentrations of fructose, acetate, and ethanol were determined by high-performance liquid chromatography (Waters) as previously described [43]. Detection was performed by UV absorption at 410 nm. Nitrate, nitrite, and ammonia concentrations were analyzed using an automated segmented flow analyzer (AA3 HR, SEAL Analytical). Ammonium concentrations were determined by flow injection analysis modified for small sample volumes [44]. The sum of nitrate and nitrite was determined spectrophotometrically after reduction of samples with cadmium [45]. The procedure was identical for determination of nitrite but without the cadmium reduction step.

### Transcriptomic profiling of *C. ljungdahlii*

The transcriptome of *C. ljungdahlii* was profiled under three different growth conditions: heterotrophically on fructose, heterotrophically on fructose with nitrate as the sole nitrogen source, and autotrophically on H_2_/CO_2_. For each of these conditions, total RNA was isolated from *C. ljungdahlii* cells growing in mid-log phase (OD_600_ of ~0.4 for both the heterotrophic conditions and an OD_600_ of ~0.09 for the autotrophic condition on H_2_/CO_2_) using the QIAGEN RNeasy Mini kit with on-column DNase I (QIAGEN) treatment. rRNA was subtracted from 2.5 μg of total RNA using the Gram-Positive Ribo-Zero rRNA removal kit (Epicentre). Then, paired-end, strand specific RNA sequencing (RNA-seq) was performed using the dUTP method [46,47] with the following changes. The rRNA subtracted RNA was fragmented with RNA fragmentation reagents (Ambion) for 3 minutes at 70°C. To synthesize first strand cDNA, random hexamer primers were used (Invitrogen). Final libraries were assayed for quality using High Sensitivity DNA chips on a Bioanalyzer (Agilent) and quantified using a Qubit (Invitrogen). A total of 12.5 pM was loaded onto a MiSeq (Illumina) to yield 31 bp paired-end reads.

The RNA-seq reads were aligned to the genome sequence of *C. ljungdahlii* (RefSeq: NC_014328.) using Bowtie [48] with two mismatches allowed per read alignment. To estimate transcript abundances, FPKM values were calculated for the protein-coding genes using Cufflinks [49] with appropriate parameters set for the strand-specific library type and upper-quartile normalization. The annotation from NCBI was used for transcript quantification. Differential expression analysis was carried out using cuffdiff, with upper-quartile normalization and appropriate parameters set for strand-specific library type. A fold change of greater than 2-fold and false discovery rate cutoff of 0.05 was used to determine significant differential expression.

## Competing interests

The authors declare that they have no competing interest.

## Authors’ contributions

HN, DRL, and KZ conceived and designed the study. HN, JN, and AE performed the reconstruction. MS and HL performed the experiments. HN carried out the simulations and analysis. HN and KZ wrote the manuscript. All authors have read and approved the manuscript.

## Supplementary Material

Additional file 1: Figure S1 Physiological growth screen of *C. ljungdahlii* grown on fructose. Shown are OD_600_, fructose, and acetate measurements. Error bars represent average of triplicate measurements. Click here for file

Additional file 2: Table S1 Flux distribution differences when *C.ljungdahlii* is simulated with and without nitrate grown on fructose. Click here for file

Additional file 3: Table S2 Differential expression of *C. ljungdahlii* grown on fructose and autotrophically on H_2_/CO_2_. Click here for file

Additional file 4: Figure S2 RNA-seq reads from transcriptome profiling of *C. ljungdahlii* during autotrophic growth on H_2_/CO_2_ showing cotranscription of MetF and MetV. Click here for file

Additional file 5 **Model 1.** Metabolic network of *C. ljungdahlii*. The model file is preset with constraints to simulate heterotrophic growth on fructose. This file can be used with COBRA toolbox in MATLAB. Click here for file

Additional file 6 **Model 2.** Metabolic network of *C. ljungdahlii*. The model file is preset with constraints to simulate autotrophic growth on H_2_/CO_2_. This file can be used with COBRA toolbox in MATLAB. Click here for file

## References

[B1] Drake HL Gössner AS Daniel SL Old acetogens, new light Ann N Y Acad Sci 2008 1125 100 128 10.1196/annals.1419.016 18378590

[B2] Nevin KP Hensley SA Franks AE Summers ZM Ou J Woodard TL Snoeyenbos-West OL Lovley DR Electrosynthesis of organic compounds from carbon dioxide Is catalyzed by a diversity of acetogenic microorganisms Appl Environ Microbiol 2011 77 2882 2886 10.1128/AEM.02642-10 21378039 PMC3126412

[B3] Nevin KP Woodard TL Franks AE Summers ZM Lovley DR Microbial electrosynthesis: feeding microbes electricity to convert carbon dioxide and water to multicarbon extracellular organic compounds MBio 2010 1 e00103 e00110 20714445 10.1128/mBio.00103-10 PMC2921159

[B4] Curtis T Daran J-M Pronk JT Frey J Jansson JK Robbins-Pianka A Knight R Schnürer A Smets BF Smid EJ Crystal ball- 2013 J Microbial Biotechnol 2013 6 3 16 10.1111/1751-7915.12014

[B5] Martin WF Hydrogen, metals, bifurcating electrons, and proton gradients: the early evolution of biological energy conservation FEBS Lett 2012 586 485 493 10.1016/j.febslet.2011.09.031 21978488

[B6] Gencic S Duin EC Grahame DA Tight coupling of partial reactions in the acetyl-CoA decarbonylase/synthase (ACDS) multienzyme complex from *Methanosarcina thermophila*: acetyl C-C bond fragmentation at the a cluster promoted by protein conformational changes J Biol Chem 2010 285 15450 15463 10.1074/jbc.M109.080994 20202935 PMC2865265

[B7] Poehlein A Schmidt S Kaster A-K Goenrich M Vollmers J Thürmer A Bertsch J Schuchmann K Voigt B Hecker M An ancient pathway combining carbon dioxide fixation with the generation and utilization of a sodium ion gradient for ATP synthesis PLoS One 2012 7 e33439 10.1371/journal.pone.0033439 22479398 PMC3315566

[B8] Buckel W Thauer RK Energy conservation via electron bifurcating ferredoxin reduction and proton/Na(+) translocating ferredoxin oxidation Biochim Biophys Acta 2012 8 94 113 22800682 10.1016/j.bbabio.2012.07.002

[B9] Lewis NE Nagarajan H Palsson BØ Constraining the metabolic genotype-phenotype relationship using a phylogeny of *in silico* methods Nat Rev Microbiol 2012 10 291 305 22367118 10.1038/nrmicro2737 PMC3536058

[B10] McCloskey D Palsson BØ Feist AM Basic and applied uses of genome-scale metabolic network reconstructions of *Escherichia coli* Mol Syst Biol 2013 9 661 23632383 10.1038/msb.2013.18 PMC3658273

[B11] Yim H Haselbeck R Niu W Pujol-Baxley C Burgard A Boldt J Khandurina J Trawick JD Osterhout RE Stephen R Metabolic engineering of *Escherichia coli* for direct production of 1,4-butanediol Nat Chem Biol 2011 7 445 452 10.1038/nchembio.580 21602812

[B12] Köpke M Held C Hujer S Liesegang H Wiezer A Wollherr A Ehrenreich A Liebl W Gottschalk G Dürre P *Clostridium ljungdahlii* represents a microbial production platform based on syngas Proc Natl Acad Sci USA 2010 107 13087 13092 10.1073/pnas.1004716107 20616070 PMC2919952

[B13] Henry CS DeJongh M Best AA Frybarger PM Linsay B Stevens RL High-throughput generation, optimization and analysis of genome-scale metabolic models Nat Biotechnol 2010 28 977 982 10.1038/nbt.1672 20802497

[B14] Lee J Yun H Feist AM Palsson BØ Lee SY Genome-scale reconstruction and in silico analysis of the *Clostridium acetobutylicum* ATCC 824 metabolic network Appl Microbiol Biotechnol 2008 80 849 862 10.1007/s00253-008-1654-4 18758767

[B15] Senger RS Papoutsakis ET Genome-scale model for *Clostridium acetobutylicum*: part I. Metabolic network resolution and analysis Biotechnol Bioeng 2008 101 1036 1052 10.1002/bit.22010 18767192 PMC2760220

[B16] Roberts SB Gowen CM Brooks JP Fong SS Genome-scale metabolic analysis of *Clostridium thermocellum* for bioethanol production BMC Syst Biol 2010 4 31 10.1186/1752-0509-4-31 20307315 PMC2852388

[B17] Milne CB Eddy JA Raju R Ardekani S Kim P-J Senger RS Jin Y-S Blaschek HP Price ND Metabolic network reconstruction and genome-scale model of butanol-producing strain *Clostridium beijerinckii* NCIMB 8052 BMC Syst Biol 2011 5 130 10.1186/1752-0509-5-130 21846360 PMC3212993

[B18] Kanehisa M Goto S KEGG: kyoto encyclopedia of genes and genomes Nucleic Acids Res 2000 28 27 30 10.1093/nar/28.1.27 10592173 PMC102409

[B19] Orth JD Palsson BØ Systematizing the generation of missing metabolic knowledge Biotechnol Bioeng 2010 107 403 412 10.1002/bit.22844 20589842 PMC3119652

[B20] Reed JL Patel TR Chen KH Joyce AR Applebee MK Herring CD Bui OT Knight EM Fong SS Palsson BØ Systems approach to refining genome annotation Proc Natl Acad Sci USA 2006 103 17480 17484 10.1073/pnas.0603364103 17088549 PMC1859954

[B21] Tanner RS Miller LM Yang D *Clostridium ljungdahlii* sp. nov., an acetogenic species in clostridial rRNA homology group I Int J Syst Bacteriol 1993 43 232 236 10.1099/00207713-43-2-232 7684239

[B22] Köpke M Mihalcea C Liew F Tizard JH Ali MS Conolly JJ Al-Sinawi B Simpson SD 2,3-Butanediol production by acetogenic bacteria, an alternative route to chemical synthesis, using industrial waste gas Appl Environ Microbiol 2011 77 5467 5475 10.1128/AEM.00355-11 21685168 PMC3147483

[B23] Campbell BJ Smith JL Hanson TE Klotz MG Stein LY Lee CK Wu D Robinson JM Khouri HM Eisen JA Cary SC Adaptations to submarine hydrothermal environments exemplified by the genome of *Nautilia profundicola* PLoS Genet 2009 5 e1000362 10.1371/journal.pgen.1000362 19197347 PMC2628731

[B24] Hasan SM Hall JB The physiological function of nitrate reduction in *Clostridium perfringens* J Gen Microbiol 1975 87 120 128 10.1099/00221287-87-1-120 166143

[B25] Ragsdale SW Pierce E Acetogenesis and the Wood-Ljungdahl pathway of CO_2_ fixation Biochim Biophys Acta Proteins Proteomics 2008 1784 1873 1898 10.1016/j.bbapap.2008.08.012 PMC2646786 18801467

[B26] Bar-Even A Does acetogenesis really require especially low reduction potential? Biochim Biophys Acta 2012 10.1016/j.bbabio.2012.1010.1007 10.1016/j.bbabio.2012.10.007 23103387

[B27] Ljungdahl LG The autotrophic pathway of acetate synthesis in acetogenic bacteria Annu Rev Microbiol 1986 40 415 450 10.1146/annurev.mi.40.100186.002215 3096193

[B28] Tremblay PL Zhang T Dar SA Leang C Lovley DR The Rnf complex of *Clostridium ljungdahlii* is a proton-translocating ferredoxin: NAD + oxidoreductase essential for autotrophic growth MBio 2012 4 e00406 e00412 23269825 10.1128/mBio.00406-12 PMC3531802

[B29] Wang S Huang H Moll J Thauer RK NADP + reduction with reduced ferredoxin and NADP + reduction with NADH are coupled via an electron-bifurcating enzyme complex in *Clostridium kluyveri* J Bacteriol 2010 192 5115 5123 10.1128/JB.00612-10 20675474 PMC2944534

[B30] Huang H Wang S Moll J Thauer RK Electron bifurcation involved in the energy metabolism of the acetogenic bacterium *Moorella thermoacetica* growing on glucose or H_2_ plus CO_2_ J Bacteriol 2012 194 3689 3699 10.1128/JB.00385-12 22582275 PMC3393501

[B31] Wang S Huang H Kahnt J Mueller AP Köpke M Thauer RK NADP-specific electron-bifurcating [FeFe]-hydrogenase in a functional complex with formate dehydrogenase in *Clostridium autoethanogenum* grown on CO J Bacteriol 2013 195 4373 4386 10.1128/JB.00678-13 23893107 PMC3807470

[B32] Wu M Ren Q Durkin AS Daugherty SC Brinkac LM Dodson RJ Madupu R Sullivan SA Kolonay JF Haft DH Life in hot carbon monoxide: the complete genome sequence of *Carboxydothermus hydrogenoformans* Z-2901 PLoS Genet 2005 1 e65 10.1371/journal.pgen.0010065 16311624 PMC1287953

[B33] Tan Y Liu J Chen X Zheng H Li F RNA-seq-based comparative transcriptome analysis of the syngas-utilizing bacterium *Clostridium ljungdahlii* DSM 13528 grown autotrophically and heterotrophically Mol Bio Syst 2013 9 2775 2784 10.1039/c3mb70232d 24056499

[B34] Schuchmann K Müller V A bacterial electron-bifurcating hydrogenase J Biol Chem 2012 287 31165 31171 10.1074/jbc.M112.395038 22810230 PMC3438948

[B35] Leang C Ueki T Nevin KP Lovley DR A genetic system for *Clostridium ljungdahlii*: a chassis for autotrophic production of biocommodities and a model homoacetogen Appl Environ Microbiol 2013 79 1102 1109 10.1128/AEM.02891-12 23204413 PMC3568603

[B36] Feist AM Palsson BØ The biomass objective function Curr Opin Microbiol 2010 13 344 349 10.1016/j.mib.2010.03.003 20430689 PMC2912156

[B37] Oh Y-K Palsson BØ Park SM Schilling CH Mahadevan R Genome-scale reconstruction of metabolic network in *Bacillus subtilis* based on high-throughput phenotyping and gene essentiality data J Biol Chem 2007 282 28791 28799 10.1074/jbc.M703759200 17573341

[B38] Xia G Kohler T Peschel A The wall teichoic acid and lipoteichoic acid polymers of *Staphylococcus aureus* Int J Med Microbiol 2010 300 148 154 10.1016/j.ijmm.2009.10.001 19896895

[B39] Feist AM Henry CS Reed JL Krummenacker M Joyce AR Karp PD Broadbelt LJ Hatzimanikatis V Palsson BØ A genome-scale metabolic reconstruction for *Escherichia coli* K-12 MG1655 that accounts for 1260 ORFs and thermodynamic information Mol Syst Biol 2007 3 121 17593909 10.1038/msb4100155 PMC1911197

[B40] Orth JD Thiele I Palsson BØ What is flux balance analysis? Nat Biotechnol 2010 28 245 248 10.1038/nbt.1614 20212490 PMC3108565

[B41] Schellenberger J Que R Fleming RMT Thiele I Orth JD Feist AM Zielinski DC Bordbar A Lewis NE Rahmanian S Quantitative prediction of cellular metabolism with constraint-based models: the COBRA toolbox v2.0 Nat Protoc 2011 6 1290 1307 10.1038/nprot.2011.308 21886097 PMC3319681

[B42] Gong Y Ebrahim A Feist AM Embree M Zhang T Lovley D Zengler K Sulfide-driven microbial electrosynthesis Environ Sci Tech 2013 47 568 573 10.1021/es303837j 23252645

[B43] Portnoy VA Herrgard MJ Palsson BØ Aerobic fermentation of D-glucose by an evolved cytochrome oxidase-deficient *Escherichia coli* strain Appl Environ Microbiol 2008 74 7561 7569 10.1128/AEM.00880-08 18952873 PMC2607145

[B44] Aminot A Kerouel R Birot D A flow injection-fluorometric method for the determination of ammonium in fresh and saline waters with a view to *in situ* analyses Water Res 2001 35 1777 1785 10.1016/S0043-1354(00)00429-2 11329680

[B45] Jones MN Nitrate reduction by shaking with cadmium: alternative to cadmium columns Water Res 1984 18 643 646 10.1016/0043-1354(84)90215-X

[B46] Parkhomchuk D Borodina T Amstislavskiy V Banaru M Hallen L Krobitsch S Lehrach H Soldatov A Transcriptome analysis by strand-specific sequencing of complementary DNA Nucleic Acids Res 2009 37 e123 10.1093/nar/gkp596 19620212 PMC2764448

[B47] Zhong S Joung J-G Zheng Y Chen Y-R Liu B Shao Y Xiang JZ Fei Z Giovannoni JJ High-throughput Illumina strand-specific RNA sequencing library preparation Cold Spring Harb Protoc 2011 2011 940 949 21807852 10.1101/pdb.prot5652

[B48] Langmead B Trapnell C Pop M Salzberg SL Ultrafast and memory-efficient alignment of short DNA sequences to the human genome Genome Biol 2009 10 R25 10.1186/gb-2009-10-3-r25 19261174 PMC2690996

[B49] Trapnell C Williams BA Pertea G Mortazavi A Kwan G van Baren MJ Salzberg SL Wold BJ Pachter L Transcript assembly and quantification by RNA-Seq reveals unannotated transcripts and isoform switching during cell differentiation Nat Biotechnol 2010 28 511 515 10.1038/nbt.1621 20436464 PMC3146043

