# Supplementary material for: Characterizing acetogenic metabolism using a genome-scale metabolic reconstruction of Clostridium ljungdahlii
Source: Microb Cell Fact. 2013 Nov 25;12:118. doi: 10.1186/1475-2859-12-118 (PMC4222884; doi:10.1186/1475-2859-12-118)
Supplement: Additional file 1: Figure S1 — Physiological growth screen of C. ljungdahlii grown on fructose. Shown are OD600, fructose, and acetate measurements. Error bars represent average of triplicate measurements. [file 1475-2859-12-118-S1.pdf]

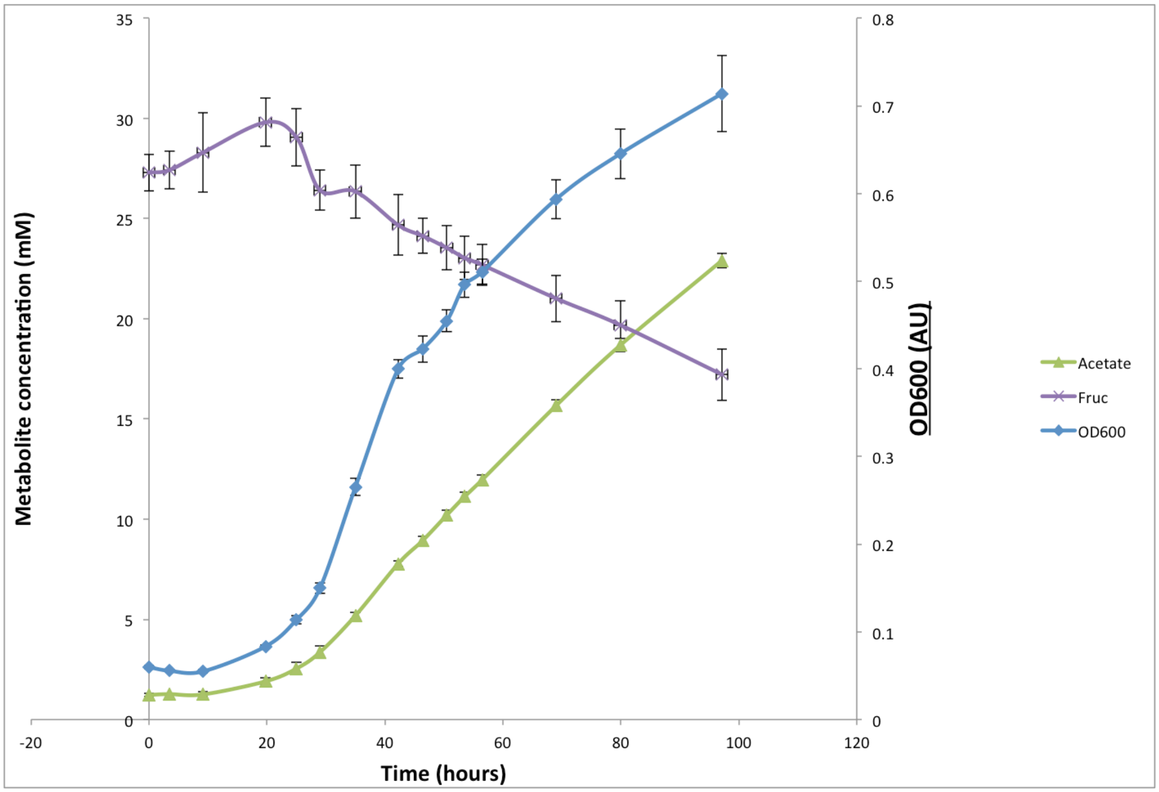

FIG S1 Physiological growth screen of *C. ljungdahlii* grown on Fructose. Shown are OD600, fructose, and acetate measurements. Error bars represent average of triplicate measurements
