# Supplementary material for: Characterizing acetogenic metabolism using a genome-scale metabolic reconstruction of Clostridium ljungdahlii
Source: Microb Cell Fact. 2013 Nov 25;12:118. doi: 10.1186/1475-2859-12-118 (PMC4222884; doi:10.1186/1475-2859-12-118)
Supplement: Additional file 4: Figure S2 — RNA-seq reads from transcriptome profiling of C. ljungdahlii during autotrophic growth on H2/CO2 showing cotranscription of MetF and MetV. [file 1475-2859-12-118-S4.pdf]

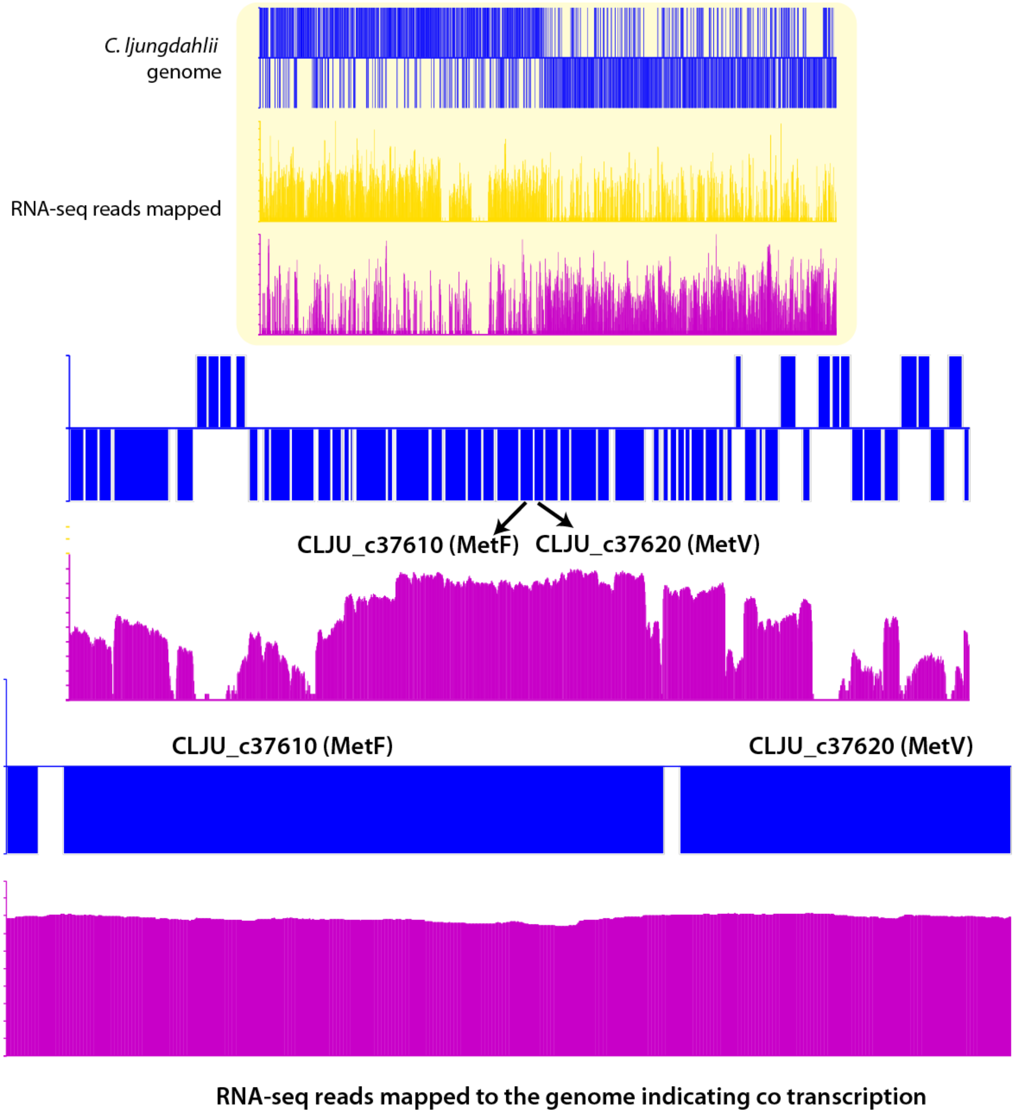

FIG S2 RNA-seq reads from transcriptome profiling of *C. ljungdahlii* during autotrophic growth on CO_2/H_2 showing cotranscription of MetF and MetV.
